# Moral Distress in Ethical Dilemmas: A Comparative Study of Medical Students and Physicians

**DOI:** 10.3390/healthcare13192547

**Published:** 2025-10-09

**Authors:** George-Dumitru Constantin, Bogdan Hoinoiu, Ioana Veja, Crisanta-Alina Mazilescu, Teodora Hoinoiu, Ruxandra Elena Luca, Ioana Roxana Munteanu, Roxana Oancea

**Affiliations:** 1 Discipline of Clinical Skills, Department I Nursing, “Victor Babeș” University of Medicine and Pharmacy Timișoara, 300041 Timișoara, Romania; george.constantin@umft.ro (G.-D.C.); tstoichitoiu@umft.ro (T.H.); 2 Doctoral School, “Victor Babeș” University of Medicine and Pharmacy Timișoara, Eftimie Murgu Square 2, 300041 Timișoara, Romania; 3 University Clinic of Oral Rehabilitation and Dental Emergencies, Faculty of Dentistry, “Victor Babeș” University of Medicine and Pharmacy Timișoara, Eftimie Murgu Square No. 2, 300041 Timișoara, Romania; hoinoiu@umft.ro (B.H.); luca.ruxandra@umft.ro (R.E.L.); munteanu.roxana@umft.ro (I.R.M.); 4 Interdisciplinary Research Center for Dental Medical Research, Lasers and Innovative Technologies, Revolutiei 1989 Avenue No. 9, 300070 Timișoara, Romania; 5 Department of Dental Medicine, Faculty of Dentistry, “Vasile Goldis” Western University of Arad, 310025 Arad, Romania; 6 Teacher Training Department, Politehnica University Timisoara, 300596 Timișoara, Romania; 7 Translational and Experimental Clinical Research Centre in Oral Health, Department of Preventive, Community Dentistry and Oral Health, Faculty of Dental Medicine, “Victor Babeș” University of Medicine and Pharmacy Timișoara, 300041 Timișoara, Romania; roancea@umft.ro

**Keywords:** ethical dilemmas, moral distress, moral reasoning, medical ethics, healthcare education, professional identity, medical ethics education, physicians, medical students

## Abstract

**Background**: Ethical dilemmas and the moral distress they generate are central challenges in healthcare practice and professional identity formation. While moral reasoning has been widely studied, comparative evidence on how medical students and practicing physicians approach ethical dilemmas remains scarce in Eastern Europe. **Methods**: A total of 244 participants (51 senior medical students and 193 physicians) completed an adapted version of the Defining Issues Test, version 2 (DIT-2). Three classical dilemmas were assessed: end-of-life decision-making, access to life-saving medication, and the reintegration of a fugitive. Responses were analyzed through descriptive statistics and chi-square tests to identify differences in decision choices and underlying reasoning. **Results**: Physicians consistently endorsed conventional, law-based reasoning, emphasizing legality and professional codes, while medical students demonstrated greater variability, indecision, and openness to compassion-driven justifications. In the “Jan and the Drug” and “Fugitive” dilemmas, significant between-group differences highlighted tensions between legality, empathy, and justice (*p* < 0.01). These differences in reasoning indicate differing vulnerabilities to moral distress, especially when legal and compassionate perspectives conflict. **Conclusions**: The findings reveal distinct patterns of moral reasoning that reflect different levels of vulnerability to moral distress in healthcare contexts. Integrating structured ethics training and reflective dialogue into both undergraduate and continuing medical education could mitigate moral distress by fostering a balance between justice, compassion, and professional responsibility.

## 1. Introduction

Moral judgment represents a cornerstone of professional identity formation in medicine and a critical determinant of ethical practice. It extends beyond the application of clinical guidelines, encompassing the ability to evaluate complex dilemmas where competing values, cultural expectations, and professional responsibilities intersect. In recent decades, research in psychology, philosophy, and bioethics has conceptualized moral reasoning as a multidimensional construct shaped by cognitive development, sociocultural context, and experiential learning [1,2,3]. From a bioethical perspective, the principles of autonomy, beneficence, justice, and non-maleficence provide an essential framework for guiding medical decision-making, yet their interpretation varies across individuals and contexts [4,5].

While these principles remain highly influential in bioethics, their universality and sufficiency have been questioned. Critics argue that principlism may be too narrow to capture the full complexity of moral decision-making, especially in diverse cultural contexts. More recent approaches, such as moral foundations theory, highlight intuitive domains including care, fairness, loyalty, authority, and sanctity [6,7], thereby offering a broader framework for interpreting ethical reasoning. Integrating these complementary perspectives enriches the conceptual basis of this study and situates our analysis within a more pluralistic understanding of morality.

Interdisciplinary approaches have enriched the study of morality by integrating insights from moral philosophy, cognitive psychology, sociology, and neuroscience. The moral foundations theory, for example, emphasizes the plurality of intuitive domains, such as care, fairness, loyalty, authority, and sanctity, that underpin moral evaluations across cultures [6,7]. This theoretical diversity underscores the need for empirical research that captures how professionals navigate dilemmas in which these moral domains conflict. Furthermore, cross-cultural studies suggest that moral judgment is not universal but contextually shaped, raising questions about how professional training and cultural background influence ethical decision-making in medicine [8,9].

Alongside moral reasoning, the concept of moral distress has gained prominence since it was first defined by Jameton in 1984 [9] as the psychological distress that arises when professionals recognize the ethically appropriate action but feel constrained from acting upon it. More recent studies have linked moral distress to burnout, diminished empathy, and compromised professional integrity in healthcare settings [10,11,12]. Ethical dilemmas in end-of-life care, distributive justice, and patient autonomy frequently trigger moral distress, highlighting the need for robust ethical reasoning skills that can help mitigate its impact.

In medical education, assessing moral reasoning provides valuable feedback for curriculum design and professional formation. The Defining Issues Test, version 2 (DIT-2), developed by Rest and Narvaez, has been widely used to measure moral judgment development across diverse populations, including medical, dental, and nursing students [13,14,15]. Previous studies have demonstrated that professional experience tends to correlate with more conventional, rule-based reasoning, while students often show variability and openness to post-conventional arguments [16,17,18]. Nevertheless, evidence from Eastern Europe remains scarce, leaving gaps in understanding how cultural context and professional identity shape ethical decision-making and vulnerability to moral distress in this region.

This study addresses these gaps by comparing moral reasoning between physicians and medical students in Romania using an adapted DIT-2. By doing so, it not only captures differences between training levels but also provides rare empirical evidence from Eastern Europe, a region underrepresented in the literature. This contextual perspective enhances the study’s relevance by extending moral reasoning research into healthcare systems where cultural and institutional dynamics may differ from those more frequently examined in Western contexts.

## 2. Materials and Methods

### 2.1. Study Design

This was a cross-sectional, observational study designed to evaluate moral judgment patterns among healthcare professionals and medical students by applying an adapted version of the Defining Issues Test, Version 2 (DIT-2). The study was conducted in accordance with the Declaration of Helsinki and was approved by the Ethics Committee of the University of Medicine and Pharmacy ‘Victor Babeș’ Timișoara (Approval No. 87/20.10.2023_rev2025).

### 2.2. Study Population

A total of 244 respondents completed the survey, comprising 51 senior medical students (20.9%) and 193 practicing physicians (79.1%). Within the student cohort, the majority were enrolled in Dentistry (82.35%) and a smaller proportion in General Medicine (17.65%). The group was predominantly female (84.31%).

The physician cohort presented a broader professional spread, with 68.39% dentists and 31.61% general practitioners. The proportion of females was 76.17%, and professional experience ranged from 1 to 28 years, with a median of 12 years (interquartile range: 6–21), ensuring representation across both early-career and senior clinicians. These characteristics are summarized in Table 1.

### 2.3. Instrument

Moral reasoning was assessed using a structured questionnaire adapted from the Defining Issues Test, Version 2 (DIT-2), originally developed by Rest and Narvaez [1]. The instrument consisted of three hypothetical but realistic moral dilemmas, each followed by a structured set of 12 statements designed to elicit different moral reasoning patterns:

- The Doctor’s Dilemma—A terminally ill patient with colon cancer requests an overdose of morphine to end her suffering. Items addressed family opinion, legal consequences, societal norms, religious values, and the physician’s personal beliefs.
- Jan and the Drug—A husband considers stealing a vital medication for his dying wife when the pharmacist refuses to reduce the price. Items focused on respect for law, intellectual property, duty to family, social justice, and the right to life.
- The Fugitive—A convict escapes after serving part of a sentence, reintegrates under a new identity, becomes successful, and contributes to society. Items explored legal punishment versus reintegration, altruism, community responsibility, and fairness.

For each dilemma, participants completed:

1. A decision task—choosing one of the proposed actions (e.g., whether to administer the overdose, steal the drug, or report the fugitive).
2. A rating task—assigning an importance score (1 = not important at all; 5 = extremely important) to each of the 12 arguments.
3. A ranking task—selecting the four most important arguments and ranking them in order.

This structure enabled the analysis of both categorical decisions and the underlying reasoning frameworks, ranging from conventional rule-based arguments to post-conventional principles of human rights and contextual judgment.

### 2.4. Instrument Reliability and Validity

The DIT-2 is a widely validated tool for moral judgment assessment, grounded in Kohlberg’s theory of moral development. The DIT-2 is a widely validated tool for moral judgment assessment, originally grounded in Kohlberg’s theory of moral development. While Kohlberg’s model has been highly influential, it has also been criticized for being overly normative, Eurocentric, and for portraying moral development in rigid stages that may obscure transitional processes. The DIT-2 reflects a neo-Kohlbergian approach that addresses these limitations by emphasizing moral schemas and the relative salience of different reasoning patterns, rather than fixed developmental stages.

It has been extensively applied in medical, dental, and nursing education research. Previous studies have reported strong internal reliability (Cronbach’s α typically ranging from 0.70 to 0.85) and solid construct validity, consistently differentiating between groups with different levels of education, professional experience, and exposure to ethics curricula [2,3].

For the present study, the dilemmas and items were translated into Romanian by two bilingual researchers, back-translated to ensure accuracy, and reviewed by a panel of three experts in bioethics and medical education. Minor wording and cultural adjustments were introduced—for example, adapting certain terms to reflect the Romanian healthcare and legal context—while preserving the original meaning and structure of the dilemmas. A pilot test with 15 participants (not included in the final analysis) confirmed clarity and comprehensibility of the items, without the need for major modifications. Thus, the term ‘adapted DIT-2’ refers specifically to this process of translation, back-translation, expert review, and cultural adjustment.

### 2.5. Procedure

The questionnaire was applied in paper format, during scheduled academic sessions (for students) and professional meetings (for physicians). Instructions emphasized that there were no “right” or “wrong” answers; rather, participants were asked to indicate their personal convictions. They were specifically reminded to:

1. Read each story carefully.
2. Choose the most appropriate course of action.
3. Rate each statement according to its importance.
4. Select and rank the four most important statements.

Completion time averaged 25–30 min. Participation was voluntary and anonymous, and respondents were not under time pressure.

### 2.6. Statistical Analysis

Data were entered into Microsoft Excel and analyzed using MedCalc Statistical Software (version 23.3.7, © 2025 MedCalc Software Ltd., Ostend, Belgium). Descriptive statistics (absolute frequencies and percentages) were calculated for all decision, rating, and ranking tasks. To test whether the distribution of Likert-scale ratings for each item differed significantly from a uniform distribution, the Chi-square goodness-of-fit test was applied. Degrees of freedom were set at 4. A *p*-value < 0.05 was considered statistically significant. Decision tasks were summarized separately, reporting proportions for each response option.

### 2.7. Ethical Considerations

The study protocol was approved by the Ethics Committee of the University of Medicine and Pharmacy “Victor Babeș” Timișoara (Approval No. 87/20.10.2023_rev2025). All participants received detailed information about the study objectives and procedures and signed informed consent prior to participation. Because moral dilemmas can occasionally provoke emotional discomfort, participants were explicitly informed of this possibility and reminded that they could skip any question or withdraw from the study at any time without consequence. Confidentiality and anonymity were strictly maintained throughout the study.

## 3. Results

### 3.1. Participant Characteristics

A total of 244 individuals initially participated in the survey, including 51 senior medical students (20.9%) and 193 practicing physicians (79.1%). Within the student cohort, most were enrolled in Dentistry (82.35%), with a smaller proportion in General Medicine (17.65%). The group was predominantly female (84.31%).

The physician cohort presented a broader professional spread, with 68.39% dentists and 31.61% general practitioners. The proportion of females was 76.17%, and professional experience ranged from 1 to 28 years, with a median of 12 years (interquartile range: 6–21), ensuring representation across both early-career and senior clinicians.

A total of 244 individuals participated in the survey. After data cleaning, 242 valid cases were retained for statistical analysis, as two participants provided incomplete or invalid responses.

These characteristics are summarized in Table 1.

### 3.2. Distribution of Decision Choices

Across the three dilemmas, response distributions indicated substantial variation in moral reasoning. In the Doctor’s Dilemma (end-of-life scenario), 38.6% of respondents supported administering a lethal overdose of morphine, 32.1% were undecided, and 29.2% rejected it. In Jan and the Drug, nearly half of respondents (48.7%) rejected theft, 30.3% were undecided, and 20.9% supported it. In The Fugitive, indecision dominated (40.8%), followed by 33.9% opposing denunciation and 25.3% supporting it. Collapsed analyses revealed no significant differences between students and physicians in the Doctor’s Dilemma (χ^2^ = 1.55, *p* = 0.461), but significant group differences in Jan and the Drug (χ^2^ = 10.12, *p* = 0.006) and The Fugitive (χ^2^ = 17.12, *p* < 0.001).

### 3.3. End-of-Life Care and Patient Autonomy

In the Doctor’s Dilemma, most arguments did not significantly differentiate groups. For instance, “The physician should respect the law, because the state has the right to force people to live” (χ^2^ = 1.46, *p* = 0.833) and “The physician should obey religious teachings, since only God decides when life ends” (χ^2^ = 8.14, *p* = 0.087) were rated similarly. Significant differences emerged in autonomy-related items: “The patient has the right to end her life when she wishes” (χ^2^ = 9.76, *p* = 0.045) and “Death can have a personal meaning beyond society’s judgment” (χ^2^ = 9.64, *p* = 0.047), with physicians rating these arguments more highly than students.

### 3.4. Conflicts Between Law, Compassion, and Property Rights

In Jan and the Drug, physicians more strongly endorsed law-based reasoning. Significant differences were found for “Laws must be respected, otherwise chaos will ensue” (χ^2^ = 13.13, *p* = 0.011), “The pharmacist has the right to set the price, since he invested in discovering the medicine” (χ^2^ = 9.77, *p* = 0.045), “Respect for law is more important than Jan’s personal situation” (χ^2^ = 10.88, *p* = 0.028), “Jan should not break the law even for love” (χ^2^ = 11.51, *p* = 0.021), and “It is dangerous to justify theft even in exceptional cases” (χ^2^ = 9.52, *p* = 0.049). Compassionate arguments such as “Love can justify theft” or “The pharmacist is cruel and immoral” did not significantly differ between groups (all *p* > 0.050).

### 3.5. Moral Judgments on Rehabilitation and Social Justice

The Fugitive dilemma revealed the strongest group divergence. Physicians endorsed law- and justice-based reasoning significantly more than students. The most striking differences were found for “Past crimes cannot be erased by later good deeds” (χ^2^ = 21.19, *p* < 0.001), “Punishment must be applied consistently, regardless of reintegration” (χ^2^ = 13.95, *p* = 0.007), and “A person must pay for their crime even if they later became a good citizen” (χ^2^ = 27.10, *p* < 0.001). Conversely, rehabilitation-oriented statements such as “The fugitive’s transformation outweighs his past crime” and “Social reintegration should be valued above punishment” showed no significant differences (all *p* > 0.050).

### 3.6. Significant Between-Group Differences at the Item Level

Overall, nine items across the three dilemmas demonstrated statistically significant differences between students and physicians. Physicians consistently rated law-based and conventional arguments as more important, while students displayed greater indecision and variability. The detailed χ^2^ and *p*-values for all items (Q1.1–Q3.12) are presented in Table 2, while the full wording of all 36 items is available Table 3. Table 2 in the main text presents a concise summary of the significant findings.

## 4. Discussion

This study explored differences in moral reasoning between medical students and physicians in Romania when confronted with three ethical dilemmas adapted from the DIT-2. The findings revealed that physicians consistently prioritized conventional, law-based arguments, whereas students displayed greater variability and indecision. These results are consistent with previous research demonstrating that professional experience tends to reinforce reliance on normative and legal frameworks, while students remain more open to post-conventional and compassion-driven reasoning [14,15,16,17,18].

It is important to note that this study did not directly measure moral distress as defined by Jameton [9], but rather moral reasoning patterns that often reflect moral uncertainty. While uncertainty differs from distress, such indecision may increase vulnerability to moral distress once individuals face real-world constraints on action. The variability and indecision observed among students suggest that they may be more vulnerable to moral distress due to the lack of a stable ethical framework, particularly when legal and compassionate perspectives are in conflict. Conversely, physicians’ stronger reliance on law-based reasoning may provide consistency but can also generate inner conflict and distress when empathy or patient-centered considerations contradict legal or institutional constraints. This dual vulnerability highlights the need to better integrate ethics training, not to eliminate such tensions, which are often irresolvable, but to help professionals navigate them more constructively. Ethics education can foster moral awareness, reflective dialogue, and resilience, equipping professionals with strategies to recognize competing demands, tolerate moral ambiguity, and maintain professional integrity despite unresolved conflict [10,11].

From an educational standpoint, these differences emphasize the importance of strengthening ethics curricula across all stages of medical training. Moral reasoning is not a static trait but a skill that develops through guided reflection, exposure to dilemmas, and interprofessional dialogue [19,20]. Teaching strategies such as case-based discussions, small-group debates, simulation scenarios, and ethics rounds have been shown to stimulate moral sensitivity and promote critical reflection [21,22,23]. Embedding such interactive methods within both undergraduate and continuing medical education could not only help students reconcile compassion with law-based reasoning but also provide physicians with opportunities to reevaluate entrenched frameworks. By fostering reflective dialogue, such strategies may reduce the risk of moral distress and promote resilience in the face of ethical challenges [12].

Beyond medical education, our findings also speak to broader issues in biomedical ethics. Healthcare systems are increasingly confronted with ethically complex situations, ranging from distributive justice and end-of-life decision-making to responsibilities during pandemics. Physicians’ preference for conventional, law-based reasoning may ensure consistency but risks underemphasizing empathy and contextual sensitivity in novel dilemmas. These results can also be interpreted through broader frameworks, such as moral foundations theory [6,7], which complement principlism and highlight the cultural and intuitive dimensions of moral reasoning. Thus, fostering a balance between normative frameworks and human-centered values is essential for preparing healthcare professionals to address emerging challenges [24,25,26,27].

The study also highlights the importance of moral reasoning for sustaining professional norms and public trust. Ethical decision-making is not solely an individual process but a collective practice that shapes professional identity and legitimacy [28]. Strengthening ethics dialogue in professional associations, policy-making, and continuous education could support more resilient standards that integrate law, compassion, and justice [29,30]. In addition, strengthening clinical ethics services and integrating trained ethics professionals into healthcare teams can provide direct support in navigating dilemmas. Such experts should be available for consultation in complex cases, while healthcare workers also need to be educated on when and how to seek this support as part of their professional development.

Limitations of the present research must be acknowledged. First, its cross-sectional design precludes conclusions about developmental trajectories of moral reasoning. Second, the sample was limited to one cultural and institutional context in Eastern Europe, which reduces generalizability but also represents a strength by addressing a gap in a region that is often overlooked in moral reasoning research. A further limitation of this study is potential selection bias, as the sample included a predominance of dentists, which may influence the generalizability of the findings. In addition, the cross-sectional design does not allow us to capture changes in moral reasoning over time. Future longitudinal studies are needed to track the development of moral reasoning across different stages of medical education and professional practice. Third, the reliance on self-reported questionnaire data may not fully capture behavior in real-world clinical practice.

Future research should adopt longitudinal designs to track moral reasoning from student years into professional life, include comparative cohorts across different cultures and professions, and integrate neuroscientific or psychological tools to complement self-report methods. Digital approaches, such as AI-supported virtual simulations or online dilemma platforms, could also provide innovative means to evaluate and enhance moral reasoning in real time [31,32,33,34].

In addition to the present findings, several complementary perspectives deserve consideration. Choi et al. have advanced the assessment of moral reasoning by validating behavioral versions of the DIT, thereby enhancing its reliability and potential for cross-cultural application [8]. Broader educational research, including the work of Slovácková [23] and Shadi et al. [33], underscores the importance of moral sensitivity and competence in nursing and allied health education, suggesting that cross-professional comparisons may yield further insights. Moreover, studies on responsible artificial intelligence and public values highlight the need to integrate technological ethics into medical curricula, as emphasized by Li et al. [35], Koehle et al. [24], Gunasekara et al. [27], Chen et al. [36], and ten Have [37]. Finally, institutional-level evidence, such as previous work on Romanian physicians’ attitudes toward business ethics [38], provides a useful baseline for longitudinal monitoring of ethical development in Eastern Europe.

## 5. Conclusions

This study demonstrated significant differences between medical students and physicians in moral reasoning when confronted with ethical dilemmas. Physicians consistently prioritized conventional, law-based arguments, while students displayed greater variability and less alignment with normative reasoning.

These findings underscore the importance of explicitly integrating ethics into both undergraduate and postgraduate curricula. At the undergraduate level, structured exposure to ethical dilemmas, guided small-group debates, and reflective writing could help students develop moral sensitivity and balance compassion with professional responsibility. For practicing physicians, continuing education programs, ethics rounds, and interprofessional workshops could provide opportunities to revisit entrenched frameworks, adapt to emerging challenges, and strengthen the ability to mediate between justice, empathy, and legal obligations. In both contexts, progressive ethics education can serve as a protective factor, reducing the risk of moral distress when professionals face irresolvable tensions between competing ethical demands.

By highlighting these differences, our study contributes to a deeper understanding of how ethical dilemmas may generate moral distress across training levels, and underscores the need for tailored strategies in healthcare education to address this challenge. Addressing moral distress through progressive ethics education is therefore essential not only for individual resilience but also for sustaining public trust, professional integrity, and the overall quality of healthcare systems.

## Figures and Tables

**Table 1:** Sociodemographic and professional characteristics of the study population.

Variable	Students (n = 51)	Physicians (n = 193)	Total (n = 244)
Gender—male, n (%)	8 (15.69)	46 (23.83)	54 (22.13)
Gender—female, n (%)	43 (84.31)	147 (76.17)	190 (77.87)
Specialization—Dentistry, n (%)	42 (82.35)	132 (68.39)	174 (71.31)
Specialization—General Medicine, n (%)	9 (17.65)	61 (31.61)	70 (28.69)
Median years of experience (IQR)	—	12 (6–21)	—

**Table 2:** **Summary of significant chi-square (χ^2^) results for individual items across the three moral dilemmas** (The Doctor’s Dilemma, Jan and the Drug, and The Fugitive). Degrees of freedom (df) were 4 for item-level analyses and 2 for collapsed decision analyses. Significant results (*p* < 0.050) are in bold.

DILEMMA	ITEM	*p*-Value
**D1**	Q1.2	0.045
**D1**	Q1.8	0.047
**D2**	Dilemma-level total	0.006
**D2**	Q2.1	0.010
**D2**	Q2.2	0.044
**D2**	Q2.7	0.027
**D2**	Q2.11	0.021
**D2**	Q2.12	0.049
**D3**	Dilemma-level total	<0.001
**D3**	Q3.1	<0.001
**D3**	Q3.4	0.007
**D3**	Q3.7	<0.001

**Notes.** Items refer to the three dilemmas adapted from the Defining Issues Test, version 2 (DIT-2). Responses were rated on a five-point Likert scale.

**Table 3:** Chi-square (χ^2^) values and significance levels (*p*) for comparisons between medical students and physicians across the three moral dilemmas. Significant results (*p* < 0.050) are highlighted in bold.

Item	Question (Item Wording)	χ^2^	df	*p*-Value
Doctor’s Dilemma (Q1.1–Q1.12)				
Q1.1	The physician should respect the law, because the state has the right to force people to live.	1.46	4	0.833
Q1.2	The patient has the right to end her life when she wishes.	9.76	4	**0.045**
Q1.3	The physician should obey religious teachings, since only God decides when life ends.	8.14	4	0.087
Q1.4	The family’s opinion must be respected, since they are directly affected.	7.32	4	0.121
Q1.5	If the physician gives the overdose, others may imitate this and society would collapse.	5.65	4	0.227
Q1.6	Death can have a personal meaning beyond society’s judgment.	9.64	4	**0.047**
Q1.7	The physician must respect professional codes and not break them.	4.32	4	0.364
Q1.8	The physician should protect the reputation of the medical profession.	6.28	4	0.179
Q1.9	Ending the patient’s life could be considered murder, which undermines justice.	7.45	4	0.114
Q1.10	Society must protect life, even if the individual wishes otherwise.	5.39	4	0.241
Q1.11	The physician should be compassionate and relieve unbearable suffering.	8.77	4	0.066
Q1.12	If the physician agrees, others may ask for the same and this would undermine medical order.	6.89	4	0.142
Jan and the Drug (Q2.1–Q2.12)				
Q2.1	Laws must be respected, otherwise chaos will ensue.	13.13	4	**0.011**
Q2.2	The pharmacist has the right to set the price, since he invested in discovering the medicine.	9.77	4	**0.045**
Q2.3	Love can justify theft, since family responsibility comes first.	4.95	4	0.293
Q2.4	Respect for law is more important than Jan’s personal situation.	10.88	4	**0.028**
Q2.5	The pharmacist is cruel and immoral for refusing to sell the medicine.	6.76	4	0.149
Q2.6	Jan should not break the law even for love.	11.51	4	**0.021**
Q2.7	Society cannot tolerate theft, regardless of motive.	5.64	4	0.228
Q2.8	It is dangerous to justify theft even in exceptional cases.	9.52	4	**0.049**
Q2.9	The right to life is more important than property rights.	6.39	4	0.172
Q2.10	The pharmacist should be sanctioned for profiting from suffering.	7.83	4	0.098
Q2.11	Jan’s theft could save his wife and justify breaking the law.	8.22	4	0.084
Q2.12	Allowing theft in this case would set a dangerous precedent for others.	4.11	4	0.392
The Fugitive (Q3.1–Q3.12)				
Q3.1	Past crimes cannot be erased by later good deeds.	21.19	4	**<0.001**
Q3.2	Punishment must be applied consistently, regardless of reintegration.	13.95	4	**0.007**
Q3.3	Reporting him would undermine his charitable contributions.	5.47	4	0.243
Q3.4	The fugitive’s transformation outweighs his past crime.	6.02	4	0.197
Q3.5	The law must be respected equally for all, without exceptions.	7.66	4	0.104
Q3.6	Denouncing him would destroy a man who rebuilt his life.	4.88	4	0.300
Q3.7	A person must pay for their crime even if they later became a good citizen.	27.10	4	**<0.001**
Q3.8	Social reintegration should be valued above punishment.	5.93	4	0.206
Q3.9	His success proves that rehabilitation can be achieved without prison.	6.78	4	0.148
Q3.10	If he is not punished, others may be encouraged to escape.	7.34	4	0.120
Q3.11	Society benefits more from his freedom than from punishment.	4.23	4	0.376
Q3.12	Protecting justice is more important than rewarding rehabilitation.	5.56	4	0.234

**Notes.** Items refer to the three dilemmas adapted from the Defining Issues Test, version 2 (DIT-2). Responses were rated on a five-point Likert scale: 1 = not important at all; 2 = of little importance; 3 = somewhat important; 4 = very important; 5 = extremely important.

## Data Availability

The original contributions presented in this study are included in the article. Further inquiries can be directed to the corresponding authors.

## Abbreviation

The following abbreviation is used in this manuscript:

DIT-2	Defining Issues Test, version 2
